# Patient reported outcomes following whole brain radiotherapy in patients with brain metastases in NSIA-LUTH Cancer Center

**DOI:** 10.1186/s12885-023-11675-8

**Published:** 2023-12-14

**Authors:** Bolanle Adegboyega, Adedayo Joseph, Adewumi Alabi, John Omomila, Lindokuhle M. Ngema, Victoria Ainsworth, Jennifer Chin, Moses O Evbuomwan, Wilfred Ngwa

**Affiliations:** 1 NSIA-LUTH Cancer Center, Lagos University Teaching Hospital, Lagos, Nigeria; 2 Johns Hopkins Medicine, Sydney Kimmel Comprehensive Cancer Centre, Baltimore, MD 21218 USA; 3 Department of Physics, University of Massachusetts Lowell, Lowell, MA 01854 USA; 4 University of Iowa Hospitals and Clinics US, Iowa City, IA 52242 USA

**Keywords:** Brain metastases, Whole-brain radiotherapy, Quality of life, Patient reported outcomes

## Abstract

**Background:**

Brain metastases (BM) are a common complication in advanced cancer patients, and extremely challenging to treat. Consequently, whole brain radiotherapy (WBRT) remains the standard palliative intervention for patients with BM. The present study set to evaluate the clinical benefits of WBRT by assessing the quality of life (QoL) in WBRT-treated patients with BM, in Nigeria.

**Methods:**

This was a prospective, longitudinal, hospital-based single-centre study. Consecutive sampling methodology was used to recruit 52 patients with BM undergoing WBRT. Patients were followed up on days 7, 30, 90 and 180 after WBRT. The EORTC QLQ-C15-PAL and EORTC QLQ-BN20 were employed to report patients’ responses. The likert scale responses were linearly converted into 0 – 100 scores, and the descriptive analysis was conducted using IBM SPSS Statistics 29.0, at 95% confidence interval, using the two-tailed* t*-test for continuous variables or the chi-square test for categorical values. The overall survival was calculated with the Kaplan Maier method and the difference tested with Log-rank method, considering the interval from the baseline until death or end of the study.

**Results:**

The study cohort was predominantly females (82.7%), and accordingly, 65.4% of the respondents had a breast primary tumor. A goodness-of-fit test yielded non-significant Chi square Pearson (*p* = 0.325) and Deviance (*p* = 1.000) residuals, indicating the best fit. The median overall survival was 180 days (~ 6 months). A total of 20 patients (38%) that survived up to 180 days reported alleviated symptoms and better functioning. A significant improvement in physical functioning (*p* < 0.001) and emotional functioning (*p* = 0.031) was reported at 180 days post WBRT, compared to baseline.

**Conclusions:**

WBRT is an effective palliative intervention in patients with BM, resulting in improved QoL. More than 50% of patients that survived ~ 3 months reported alleviation of pain, and 38% of patients that survived for ~ 6 months reported a significantly improved functioning. This demonstrated the clinical benefits of WBRT in palliative care and will add to the body of data on the use of WBRT, from Africa.

**Supplementary Information:**

The online version contains supplementary material available at 10.1186/s12885-023-11675-8.

## Background

Brain metastases (BM) are a common occurrence in patients with advanced cancers, accounting for about 25 – 50% of intracranial tumors [1, 2]. BM emanate from the spread of cancer cells from a primary site in the body to the brain, and often outnumber primary brain tumors [3]. Particularly, the lungs and breasts are the most common primary sites from which BM may originate [4]. It is estimated that approximately 20 – 40% of advanced cancer patients, develop BM in the course of their illness [5], resulting in extensive morbidity and limited life expectancy [1]. Accordingly, the treatment of patients presenting with BM continues to be a daunting challenge as there is no definite cure [4].

Whole-brain radiotherapy (WBRT) is the current standard intervention for patients with BM who are not favourable to undergo surgery or stereotactic radiotherapy [5, 6]. Essentially, WBRT is a palliative intervention aimed at improving neurological deficits while preventing further decline in neurological function [6, 7]. Various reports indicate that the response rate of WBRT ranges from 40 – 60%, although potential side effects including nausea, hair loss, fatigue, and neuro-cognitive deficits may be inevitable [1, 5, 8, 9]. Owing to the side effects, patient survival may be shortened following WBRT, and this may cause controversy on whether the treatment should be offered or not [1].

Quality of life (QoL) is one of the key endpoints in oncological clinical investigations, essential for assessing the clinical benefits of different treatment options. Various studies have reported contrasting results of QoL measurements in patients with BM receiving WBRT treatment [10–12], and these may be due to varying fractionation schemes, assessment time points, as well as patient cohorts [13]. Generally, patients with BM present with poor QoL at the onset, and relatively short survival periods [9]. Therefore, it is necessary to define whether the WBRT treatment is beneficial and which prognostic factors are favourable in this regard. Accordingly, the purpose of the present study was to investigate the clinical benefits of WBRT by assessing the QoL and related survival outcomes reported by the patients with BM, following WBRT treatment. The patients’ characteristics and factors associated with improving and/or declining QoL status, as well as worsening of BM related symptoms were analyzed.

## Methods

### Patient recruitment

A total of 52 patients presenting with BM were recruited and gave informed consent to be part of a prospective, longitudinal, hospital-based single-center study carried out at Nigeria Sovereign Investment Authority (NSIA) - Lagos University Teaching Hospital (LUTH) Cancer Center. The study received ethical approval (ADM/DCST/HREC/APP/3558) from the Ethics Committee, on the 13^th^ of March 2020. A consecutive sampling methodology was employed for patient selection and inclusion. Demographic details such as age, gender, employment status, religion, marital status, and occupation, as well as clinical records including primary tumor location, presence of co-morbidities, ECOG performance status, prior radiotherapy (RT), date of diagnosis of BM, and number and volume of BM were attained, prior to treatment [14]. Patients received 20 Gy in 5 fractions (48%) and 30 Gy in 10 fractions (52%) of WBRT regimen and were followed up on days 7, 14, 30, 90, and 180 post treatment.

### Treatment outcome scoring and reporting

Standardized European Organization for Research and Treatment of Cancer Quality of Life Questionnaire-Core-15-Palliative Care (EORTC QLQ-C15-PAL) and EORTC QLQ Brain Neoplasm Questionnaire (QLQ-BN20) follow-up forms were used to score treatment outcomes. All patients were asked to complete the forms independently at different times of evaluation (i.e., at the time of presentation and following WBRT), with the help of the interpreter for illiterate patients. The EORTC QLQ-C15-PAL is an abbreviated tool to assess QoL in patients treated palliatively, which contains 15 items, with 2 functional scales (i.e., physical and emotional) and symptom scale [15]. Meanwhile, EORTC QLQ-BN20 is a validated questionnaire for patients with primary brain tumors, often used for patients with BM to supplement the QLQ-C15-PAL. It comprises 20 questions scored as 4 multi-item functional scales (i.e., future uncertainty, visual disorder, motor dysfunction, and communication deficit), and 7 single-item symptom scales (i.e., headache, seizures, drowsiness, hair loss, itchy skin, weakness of legs, and bladder control) [15, 16]. The self-assessed responses were recorded in a 4-likert scale, and linearly converted to a 0 – 100 scale, with higher scores in symptoms indicating severity, whereas in the case of functions, higher scores indicated better function.

### Statistical analysis

The raw scores for EORTC QLQ-C15-PAL and EORTC QLQ-BN20 were computed and linearly transformed to a 0 – 100 scale. All variables and score transformation procedure are presented in Additional file 1 (Table S1) and Additional file 2, respectively. The transformed scores of all items were expressed as arithmetic means and standard deviations, at 95% confidence interval (*p* < 0.05). A descriptive analysis was duly performed, with *p* values calculated using the two-tailed t-test for continuous variables or the chi-square test for categorical values, with *p* < 0.05 considered statistically significant. The normality tested using the Shapiro–Wilk test, and the goodness-of-fit determined from the Chi square Pearson and Deviance residuals. The overall survival was calculated with the Kaplan Maier method and the difference tested with Log-rank method, considering the interval from the baseline until death or end of the study. The data was analyzed using IBM SPSS Statistics 29.0.

## Results

The present study involved 52 patients with BM, recruited on presentation to NSIA-LUTH and followed-up after receiving WBRT treatment. Presented in Table 1 is the summary of the socio-demographic and clinical data collected from the recruited patients. On average, the patients were ~ 53 years old and predominantly female (82.7%). The majority (90.4%) of the patients were originally from or based in the urban areas, married (80.8%), and had post-secondary education (82.7%). Breast, lung, and head and neck were the most reported primary tumor types, making up 65.4%, 13.5%, and 5.3% of the cases, respectively. Although, 27% of the cohort presented with a Karnofsky performance status (KPS) above 60, a 46.1% largely presented with a KPS below 50. Additionally, more than at least half (59.6%) of the patients presented with co-morbidities, and 92.3% were already on steroids. A 36.5% had received radiotherapy to other sites, meanwhile 63.5% had no prior radiotherapy treatments.

**Table 1:** Patient socio-demographics and clinical data (*n* = *52,* enrolled patients)

Variable	Total
**Age** (mean ± SD)	53 ± 12.3
**Sex**	
Male	9 (17.3%)
Female	43 (82.7%)
**Place of residence**	
Urban	47 (90.4%)
Rural	5 (9.6%)
**Level of education**	
Secondary	9 (17.3%)
Post-secondary	43 (82.7%)
**Marital status**	
Single	5 (9.6%)
Married	42 (80.8%)
Divorced	1 (1.9%)
Widow	4 (7.7%)
**Occupation**	
Employed	31 (59.6%)
Unemployed	15 (28.8%)
Self-employed	5 (9.6%)
Retired	1 (1.9%)
**Primary tumor**	
Anaplastic thyroid	1 (1.9%)
Breast	34 (65.4)
Carcinoid tumor	1 (1.9%)
Cup	1 (1.9%)
GI	1 (1.9%)
Head and neck	3 (5.8%)
LT thigh	1 (1.9%)
Lungs	7 (13.5%)
Parotid	1 (1.9%)
Prostate	1 (1.9%)
Renal	1(1.9%)
**ECOG performance status**	
KPS 90 – 100	7 (13.5%)
KPS 70 – 80	7 (13.5%)
KPS 50 – 60	14 (26.9%)
KPS 30 – 40	13 (25.0%)
KPS 10 – 20	11 (21.1%)
KPS 0	-
**Prior radiotherapy**	
Yes	19 (36.5%)
No	33 (63.5%)
**Co-morbidities**	
Present	31 (59.6%)
Absent	21 (40.4%)
**Medication used**	
Steroids	48 (92.3%)
Analgesic	2 (3.8%)
Analgesic/steroids	2 (3.8%)

*GI* Gastrointestinal, *LT thigh* left thigh sarcoma, *KPS* Karnofsky performance status

The descriptive analysis output for both EORTC QLQ-C15-PAL and EORTC QLQ-BN20 subscales are presented in Table 2 and Table 3, respectively. Accordingly, it was revealed that the subscales were not normally distributed (sig. *p* < 0.05), however, the Shapiro–Wilk statistic (W) closer to 1 suggested a good fit. A goodness-of-fit test ascertained the assumption, yielding non-significant Chi square Pearson (*p* = 0.325) and Deviance (*p* = 1.000) residuals, indicating that the model fit the data well.

**Table 2:** Descriptive statistics for the EORTC QLQ-C15-PAL

	**Period**	**No. patients (N)**	**Mean (SD)**	**Lower bound**	**Upper bound**	**Normality (Shapiro–Wilk, *W*)**
Physical functioning	Baseline	52	2.5 (1.1)	2.2	2.8	0.87
	Day 7	49	2.2 (1.0)	1.9	2.5	0.89
	Day 30	46	2.1 (1.0)	1.8	2.4	0.88
	Day 90	28	1.8 (0.8)	1.4	2.1	0.85
	Day 180	20	1.8 (0.9)	1.4	2.2	0.86
Emotional functioning	Baseline	52	1.6 (0.8)	1.4	1.9	0.74
	Day 7	49	1.4 (0.7)	1.2	1.6	0.71
	Day 30	46	1.6 (1.7)	1.1	2.1	0.39
	Day 90	28	1.2 (0.4)	1.1	1.4	0.56
	Day 180	20	1.2 (0.3)	1.0	1.3	0.58
Fatigue	Baseline	52	2.1 (0.8)	1.9	2.3	0.90
	Day 7	49	1.9 (0.8)	1.7	2.1	0.89
	Day 30	46	1.7 (0.7)	1.5	2.0	0.82
	Day 90	28	1.9 (0.6)	1.7	2.1	0.91
	Day 180	20	1.8 (0.4)	1.6	2.0	0.87
Pain	Baseline	52	2.2 (0.9)	2.0	2.5	0.92
	Day 7	49	2.0 (0.8)	1.8	2.2	0.92
	Day 30	46	1.9 (0.7)	1.6	2.1	0.88
	Day 90	28	1.8 (0.7)	1.5	2.1	0.88
	Day 180	20	1.9 (0.6)	1.6	2.2	0.90
Dyspnea	Baseline	52	1.3 (0.6)	1.2	1.5	0.57
	Day 7	49	1.2 (0.4)	1.1	1.3	0.45
	Day 30	46	1.1 (0.4)	1.0	1.3	0.41
	Day 90	28	1.2 (0.4)	1.0	1.3	0.47
	Day 180	20	1.2 (0.5)	1.0	1.4	0.45
Insomnia	Baseline	52	2.1 (0.9)	1.8	2.4	0.86
	Day 7	49	1.9 (0.8)	1.6	2.1	0.82
	Day 30	46	1.8 (0.8)	1.6	2.1	0.81
	Day 90	28	1.6 (0.6)	1.4	1.9	0.76
	Day 180	20	2.1 (0.5)	1.8	2.3	0.69
Appetite loss	Baseline	52	1.9 (0.9)	1.6	2.1	0.81
	Day 7	49	1.5 (0.6)	1.3	1.7	0.72
	Day 30	46	1.5 (0.8)	1.3	1.7	0.68
	Day 90	28	1.5 (0.6)	1.3	1.8	0.73
	Day 180	20	1.7 (0.7)	1.4	2.0	0.78
Nausea	Baseline	52	1.4 (0.7)	1.2	1.6	0.60
	Day 7	49	1.3 (0.6)	1.1	1.4	0.49
	Day 30	46	1.2 (0.5)	1.1	1.4	0.59
	Day 90	28	1.1 (0.3)	1.0	1.2	0.36
	Day 180	20	1.3 (0.5)	1.1	1.5	0.58
Constipation	Baseline	52	1.6 (0.8)	1.3	1.8	0.67
	Day 7	49	1.5 (0.9)	1.3	1.8	0.67
	Day 30	46	1.2 (0.6)	1.1	1.4	0.46
	Day 90	28	1.1 (0.5)	0.9	1.3	0.29
	Day 180	20	1.1 (0.2)	1.0	1.2	0.24

*W* Shapiro–Wilk test statistic

**Table 3:** Descriptive statistics for the EORTC QLQ-BN20

	**Period**	**No. patients (N)**	**Mean (SD)**	**Lower bound**	**Upper bound**	**Normality (Shapiro–Wilk, *W*)**
Future uncertainty	Baseline	52	1.8 (0.7)	1.6	2.1	0.87
	Day 7	49	1.7 (0.6)	1.5	1.8	0.86
	Day 30	46	1.5 (0.6)	1.4	1.7	0.81
	Day 90	28	1.5 (0.7)	1.3	1.8	0.79
	Day 180	20	1.7 (0.6)	1.4	2.0	0.90
Visual disorder	Baseline	52	1.6 (0.7)	1.4	1.8	0.79
	Day 7	49	1.5 (0.5)	1.3	1.6	0.82
	Day 30	46	1.5 (0.6)	1.3	1.7	0.79
	Day 90	28	1.3 (0.4)	1.1	1.5	0.72
	Day 180	20	1.4 (0.5)	1.1	1.6	0.76
Motor dysfunction	Baseline	52	1.9 (0.8)	1.7	2.2	0.89
	Day 7	49	1.8 (0.7)	1.6	2.1	0.92
	Day 30	46	1.8 (0.7)	1.6	2.0	0.91
	Day 90	28	1.5 (0.4)	1.4	1.7	0.80
	Day 180	20	1.6 (0.5)	1.3	1.8	0.90
Communication deficit	Baseline	52	1.5 (0.8)	1.3	1.7	0.68
	Day 7	49	1.3 (0.5)	1.1	1.5	0.63
	Day 30	46	1.3 (0.6)	1.1	1.4	0.52
	Day 90	28	1.2 (0.4)	1.0	1.3	0.51
	Day 180	20	1.1 (0.2)	1.0	1.1	0.45
Headache	Baseline	52	2.4 (1.1)	2.5	2.8	0.85
	Day 7	49	1.7 (0.8)	1.4	1.9	0.77
	Day 30	46	1.4 (0.6)	1.2	1.5	0.64
	Day 90	28	1.2 (0.4)	1.0	1.3	0.47
	Day 180	20	1.4 (0.6)	1.1	1.6	0.63
Seizures	Baseline	52	1.4 (0.7)	1.2	1.6	0.58
	Day 7	49	1.2 (0.5)	1.1	1.4	0.52
	Day 30	46	1.3 (0.7)	1.5	1.5	0.53
	Day 90	28	1.1 (0.3)	1.0	1.2	0.29
	Day 180	20	1.1 (0.3)	1.0	1.2	0.35
Drowsiness	Baseline	52	1.6 (0.8)	1.3	1.8	0.70
	Day 7	49	1.5 (0.6)	1.3	1.6	0.69
	Day 30	46	1.4 (0.7)	1.1	1.6	0.58
	Day 90	28	1.2 (0.5)	1.0	1.4	0.54
	Day 180	20	1.2 (0.4)	1.0	1.3	0.43
Hair loss	Baseline	52	1.1 (0.4)	1.0	1.2	0.34
	Day 7	49	1.2 (0.6)	1.0	1.3	0.34
	Day 30	46	1.2 (0.5)	1.0	1.3	0.36
	Day 90	28	1.1 (0.3)	1.0	1.2	0.29
	Day 180	20	1.0 (0.0)	1.0	1.0	-
Itchy skin	Baseline	52	1.1 (0.3)	1.0	1.2	0.40
	Day 7	49	1.1 (0.4)	1.0	1.3	0.31
	Day 30	46	1.1 (0.4)	1.0	1.2	0.32
	Day 90	28	1.0 (0.2)	1.0	1.1	0.19
	Day 180	20	1.1 (0.2)	1.0	1.2	0.24
Weakness of both legs	Baseline	52	1.9 (0.9)	1.6	2.1	0.78
	Day 7	49	1.8 (1.0)	1.6	2.1	0.78
	Day 30	46	1.7 (0.9)	1.5	2.0	0.76
	Day 90	28	1.3 (0.5)	1.1	1.5	0.59
	Day 180	20	1.3 (0.4)	1.0	1.5	0.54
Bladder control	Baseline	52	1.8 (0.9)	1.6	2.1	0.78
	Day 7	49	0.6 (0.8)	1.4	1.8	0.71
	Day 30	46	1.4 (0.8)	1.2	1.7	0.63
	Day 90	28	1.3 (0.5)	1.1	1.5	0.58
	Day 180	20	1.3 (0.6)	1.0	1.5	0.52

*W* Shapiro–Wilk test statistic

Presented in Table 4 and Table 5 are the results of the multi-trait scaling analyses for EORTC QLQ-C15-PAL and EORTC QLQ-BN20, respectively. A Spearman’s correlation test showed that there was a significant correlation (*p* < 0.01) between all items in the EORTC QLQ-C15-PAL, meanwhile in the EORTC QLQ-BN20, there was a significant correlation (*p* < 0.01) between future uncertainty, motor dysfunction, and communication deficit, as well as between visual disorder and communication deficit (*p* < 0.05).

**Table 4:** EORTC QLQ-C15-PAL multi-trait scaling correlation

		**PF**	**EF**	**FA**	**PA**
**Physical functioning (PF)**	*ρ*	1.000	0.367**	0.628**	0.581**
	*Sig*	-	0.008	< 0.001	< 0.001
**Emotional functioning (EF)**	*ρ*	0.367**	1.000	0.606**	0.468**
	*Sig*	0.008	-	< 0.001	< 0.001
**Fatigue (FA)**	*ρ*	0.628**	0.606**	1.000	0.554**
	*Sig*	< 0.001	< 0.001	-	< 0.001
**Pain (PA)**	*ρ*	0.581**	0.468**	0.554**	1.000
	*Sig*	< 0.001	< 0.001	< 0.001	-

*ρ* Spearman’s rank correlation coefficient, *sig.* statistical significance (**correlation is significant at the 0.01 level, two-tailed)

**Table 5:** EORTC QLQ-BN20 multi-trait scaling correlation

		**FU**	**VD**	**MD**	**CD**
**Future uncertainty (FU)**	*ρ*	1.000	0.241	0.406**	0.422**
	*Sig*	-	0.089	0.003	0.002
**Visual disorder (VD)**	*ρ*	0.241	1.000	0.190	0.310*
	*Sig*	0.086	-	0.17	0.026
**Motor dysfunction (MD)**	*ρ*	0.406**	0.190	1.000	0.327*
	*Sig*	0.003	0.178	-	0.018
**Communication deficit (CD)**	*ρ*	0.422**	0.310*	0.327*	1.000
	*Sig*	0.002	0.026	0.018	-

*ρ* Spearman’s rank correlation coefficient, *sig.* statistical significance (**correlation is significant at the 0.01 level, *correlation is significant at the 0.05 level, two-tailed)

**Fig. 1:**
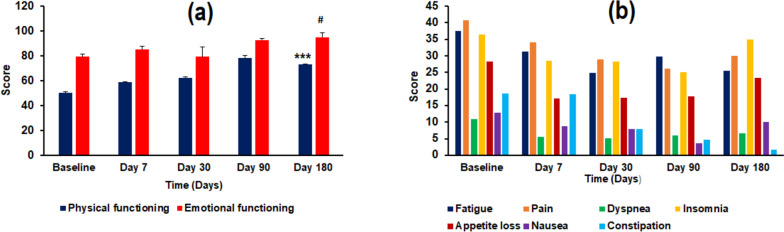
A representation of (**a**) physical and emotional functioning scores with corresponding (**b**) symptom scores, as reported by the patients in an EORTC QLQ-C15-PAL overtime. The self-assessed scores indicated improved functionality (higher scores) and symptom alleviation (lower scores) following WBRT treatment. (***depicts *p* < 0.001, # depicts *p* < 0.05, compared to baseline)

The transformed functional and symptom scores from the EORTC QLQ-C15-PAL are depicted in Fig. 1. An increase in functional (i.e., physical and emotional functioning) scores was recorded over time, from baseline till day 180 (end of study), indicating improvement in functioning following WBRT (Fig. 1a). However, physical functioning started to decline after 90 days. Nonetheless, the recorded scores for both physical functioning and emotional functioning at the end of the study were significantly higher (*p* < 0.001; *p* < 0.05) than the recorded scores at baseline. A decline in scores was recorded for symptoms, which indicated alleviation overtime (Fig. 1b). However, after 90 days, the scores started to increase (as seen on day 180), indicating the worsening of the symptoms. The raw and transformed scores from EORTC QLQ-C15-PAL and EORTC QLQ-BN20, are provided in Additional file 3.

**Fig. 2:**
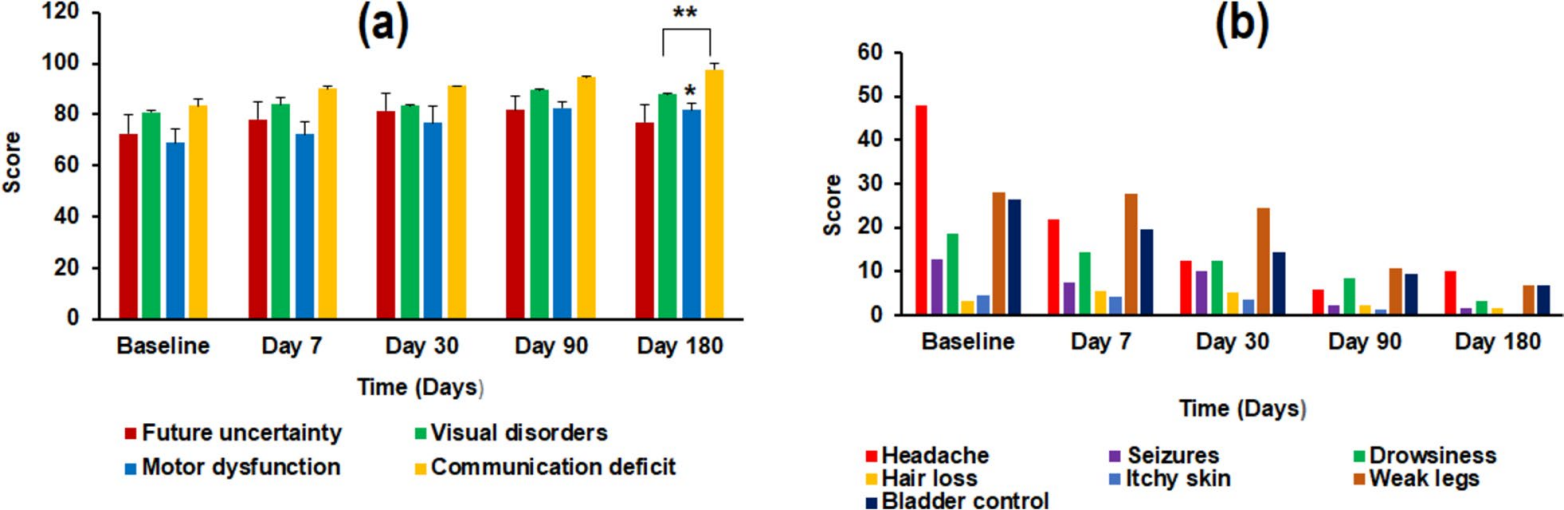
Self-assessed (**a**) functionality scores and (**b**) symptom scores, as reported by the patients in an EORTC QLQ-BN20 overtime. Improved functionality (higher scores) and symptom alleviation (lower scores) was reported by the patients following WBRT treatment. (* depicts *p* < 0.05; **depicts *p* < 0.01, compared to baseline)

Shown in Fig. 2 are the transformed self-assessed functional (i.e., future uncertainty, visual disorders, motor dysfunction, and communication deficit) scores (Fig. 2a), and the symptom scores (Fig. 2b) for EORTC QLQ-BN20. As observed in the EORTC QLQ-C15-PAL responses, a similar trend in functionality was observed in EORTC QLQ-BN20, with increasing functionality scores over time (until day 90). Meanwhile a continuous decline in symptom scores (corresponding to alleviation) over time until end of study (day 180) was reported, with the exception of headache, for which the score increased on day 180.

**Fig. 3:**
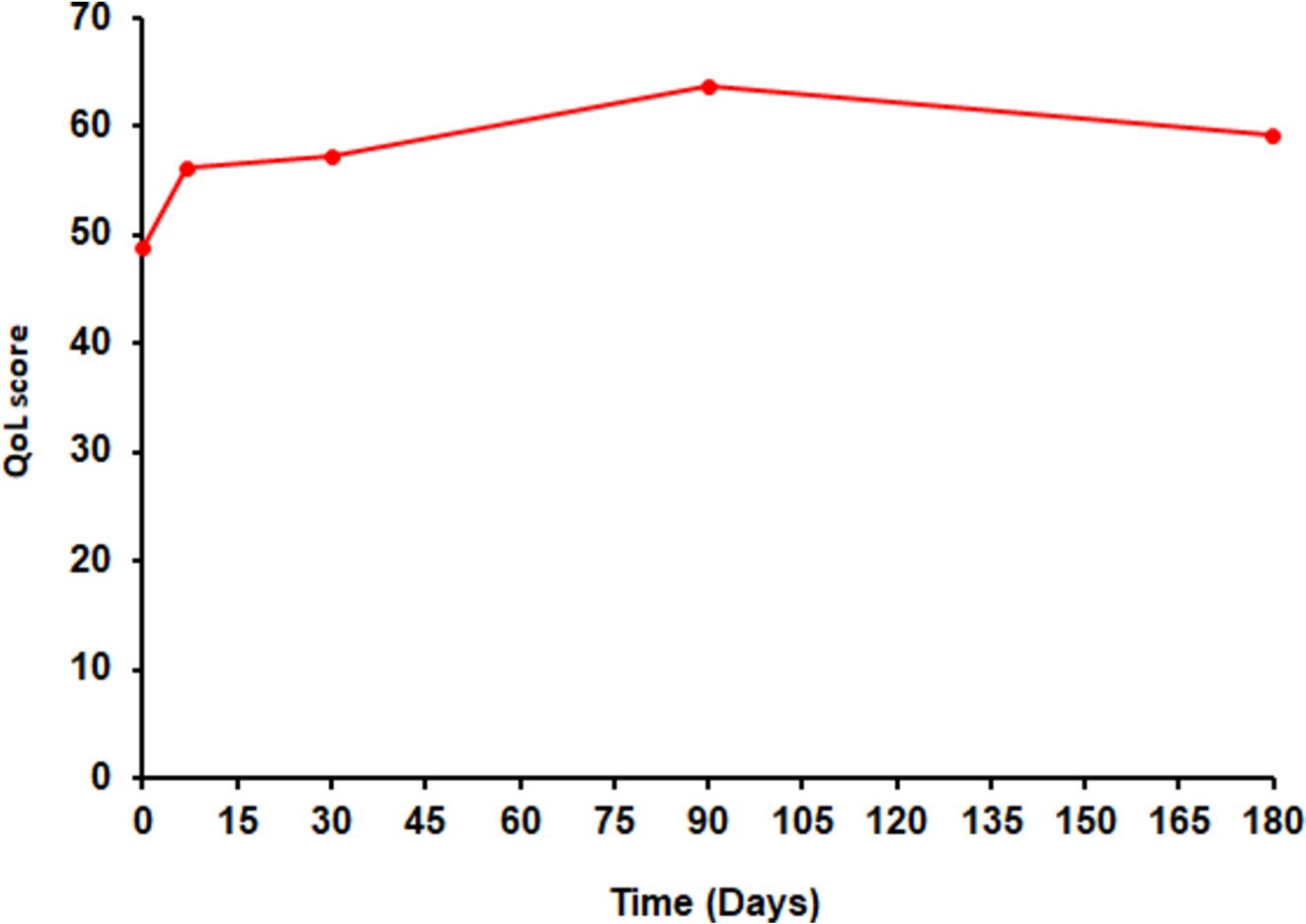
QoL measurement in patients presenting with BM, following treatment with WBRT. QoL gradually increased until day 90, and started declining at day 180 (end of study)

Depicted in Fig. 3 is the QoL status following WBRT treatment. Patients reported a gradual improvement in the QoL, with the highest positive responses recorded on day 90 post WBRT. Similarly to the responses recorded in EORTC QLQ-C15-PAL, patients also reported a decline in the QoL after 90 days, which corresponds to the reported decline in physical and emotional functioning, as well as symptom aggravation after 90 days.

**Fig. 4:**
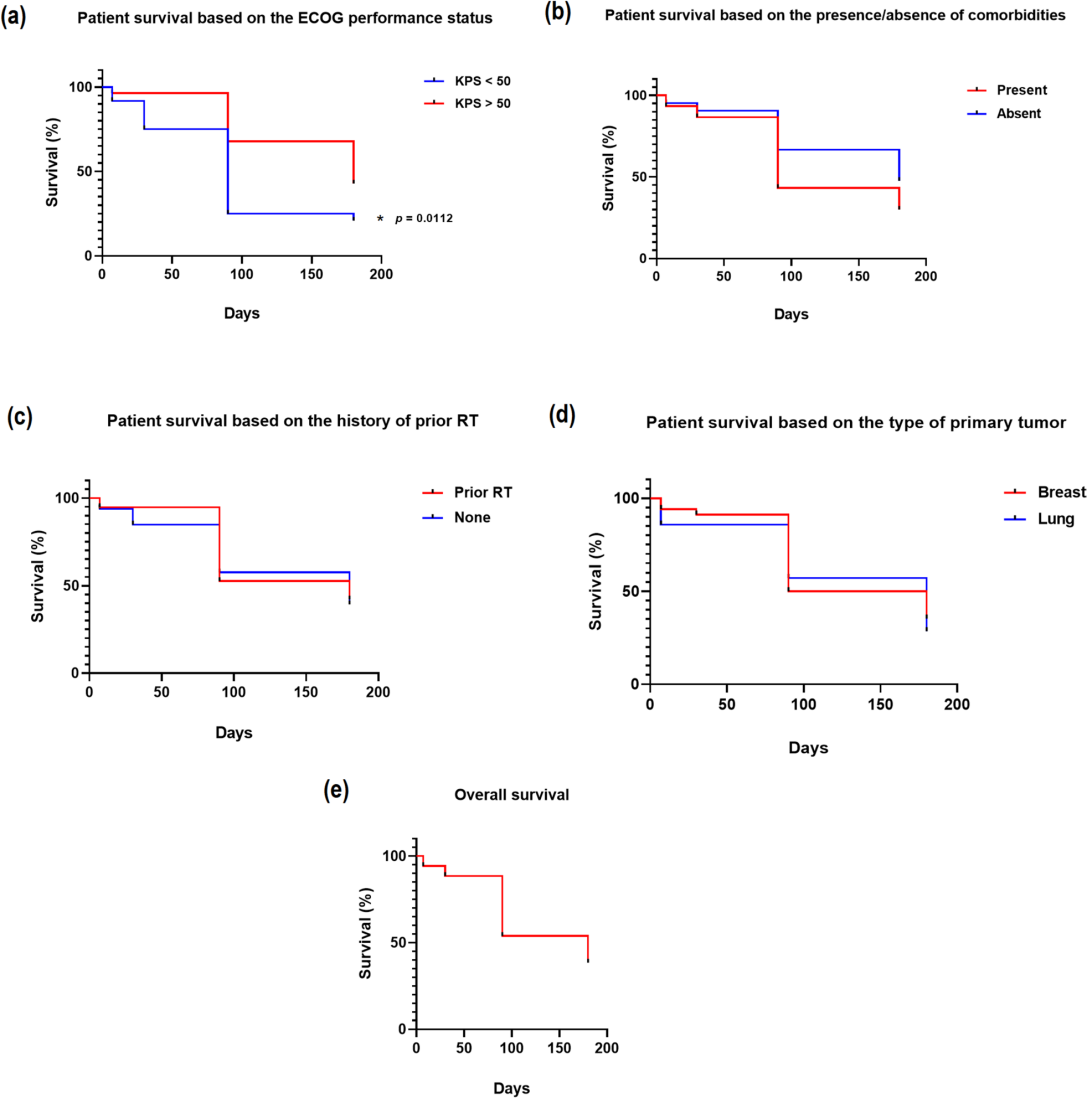
Kaplan–Meier survival curves for predicted prognostic factors; (**a**) ECOG performance status, (**b**) co-morbidities, (**c**) prior RT, and (**d**) type of primary tumor, as well as (**e**) overall survival. Patients with a KPS < 50 exhibited significantly lower survival opportunities compared to their counterparts with a KPS > 50. A 38.5% of the cohort survived till end of study, and the median overall survival was 180 days

The Kaplan–Meier curves of the prognostic factors known to influence patient survival in palliative setting, and possibly affect the clinical benefits of WBRT treatment [1, 5, 13], are presented in Fig. 4a, b, c, d. Also presented in Fig. 4e is the overall survival curve. The KPS score was found to be the only factor that significantly affected patient survival, with patients presenting with a KPS < 50 demonstrating a significantly lower (*p* = 0.0112) survival percentage than patients with a KPS > 50. Although patients that presented with co-morbidities exhibited a comparatively lower survival percentage than patients without co-morbidities, the difference was not statistically significant. Likewise, the comparison based on the type of primary tumor as well as the history of prior RT did not show any statistical significance in patient survival. Out of 52 patients recruited for the study, 20 survived till end of study (180 days), and the % survival overtime was; day 7 = 94.2%, day 30 = 88.5%, day 90 = 53.8%, and day 180 = 38.5%. The median overall survival was found to be 180 days (~ 6 months).

## Discussion

In this study, we have used the standard QoL measurement tools, the EORTC QLQ-C15-PAL and EORTC QLQ-BN20, to evaluate the patient reported outcomes following WBRT treatment. Accordingly, the responses, in the form of self-assessed scores from 52 responders were analysed to deduce sound conclusions on the clinical benefit of WBRT. Essentially, the descriptive analysis results showed that the obtained data was representative of the clinical sample/population, as seen from other related reports [1, 14], alluding to the robustness of the employed EORTC questionnaires. Improvement in functioning and alleviation of symptoms was reported from the first week of follow-up, with subsequent positive responses through 180 days (~ 6 months). It is believed that the alleviation of symptoms lead to the improvement of functionality, and this is further supported by the defined correlation between these two factors, as demonstrated in the multi-trait scaling analyses.

Alleviation of pain, headache, insomnia and bladder control were the most common benefits reported by the patients, at least up to 90 days (~ 3 months) after WBRT. Several studies have also reported reduced headache and improvement of insomnia as the common benefits reported by the patients with BM after 1 – 3 months of receiving WBRT [10, 17–19]. In addition, alleviation of fatigue was also reported by the patients in the present study for up to 30 days (~ 1 month) after WBRT, however, it aggravated thereafter. A similar occurrence has been reported, where fatigue was examined in 288 patients after WBRT, and it was found that after 8 weeks (~ 2 months), fatigue significantly worsened [20]. Primarily, these findings concur with the reports that have defined fatigue as one of the major side-effects of WBRT [1, 5, 10].

A steady improvement in the QoL status was reported over time, up to 90 days (~ 3 months), which then declined thereafter. The global health/QoL score was 48 at baseline and increased to 63 at 90 days (~ 3 months), before a decline to 59 at 180 days (~ 6 months). Nonetheless, this showed a relative improvement in QoL over time, suggestive of the positive benefits of WBRT. A related study reported a global QoL score of 52 at baseline in patients presenting with BM, which deteriorated to 42 at 3 months [1], with more other studies reporting a decline in QoL at 3 months [13, 21]. All these studies report a decline in the QoL scores at 2 or 3 months, however, in the present study the patients reported an improved QoL at 3 months compared to baseline, and it was only after 3 months that a decline in the QoL was noted. It is believed that improved functioning and symptom alleviation culminated in improved QoL.

A significant difference in survival based on the KPS score is reported in the present study, with patients with a KPS > 50 exhibiting a prolonged survival compared to those with a KPS < 50. Other predicted prognostic factors (i.e., co-morbidities, prior RT, and primary tumor type) did not show any statistical significance in influencing patient survival. A relatively prolonged overall survival, with a median of 180 days (~ 6 months) was found in the study. Meanwhile, one other study had previously reported a median overall survival of 3.5 months in a cohort of 173 patients with BM, treated with WBRT, and indicated that 43 of the patients died within the first 2 months and those that survived thereafter had a median survival of 8.1 months [1]. In essence, our findings indicate that KPS remains a significant prognostic factor affecting patient survival.

## Conclusions

The present prospective study has evaluated the clinical benefits of WBRT in patients with BM, and indicated that improvement in both physical and emotional functioning, as well as alleviation of common symptoms such as pain, headache, and insomnia are the most noted benefits of WBRT by the patients over time. These benefits could be further associated with the improvement in the overall QoL reported after WBRT treatment. Moreover, WBRT was found to result in a median overall survival of 6 months, meanwhile, a KPS score below 50 (KPS < 50) was found to be an unfavourable prognostic factor, significantly affecting patient survival and potentially limiting the clinical benefits of WBRT. The data from this study will add to the current knowledge regarding the use of WBRT in palliative care, particularly in Africa, and its compelling clinical benefits. More studies are set to be conducted in which the EORTC questionnaires are translated into preferred native languages.

### Supplementary Information

**Additional file 1: Table S1.** Brain metastasis variable key.

**Additional file 2. **Score transformation procedure.

**Additional file 3. **Raw and transformed patient reported scores.

## Data Availability

The dataset supporting the conclusions of this article is included within the article (and its additional files).

## Abbreviations

BM: Brain metastases
EORTC QLQ-C15-PAL: European Organization for Research and Treatment of Cancer Quality of Life Questionnaire-Core-15-Palliative Care
EORTC QLQ-BN20: European Organization for Research and Treatment of Cancer Quality of Life Brain Neoplasm Questionnaire
KPS: Karnofsky performance status
OS: Overall survival
QoL: Quality of life
WBRT: Whole brain radiotherapy
