# Supplementary material for: Patient reported outcomes following whole brain radiotherapy in patients with brain metastases in NSIA-LUTH Cancer Center
Source: BMC Cancer. 2023 Dec 14;23:1233. doi: 10.1186/s12885-023-11675-8 (PMC10722749; doi:10.1186/s12885-023-11675-8)
Supplement: Supplementary file 1 — Additional file 1: Table S1. Brain metastasis variable key. [file 12885_2023_11675_MOESM1_ESM.docx]

Patient reported outcomes following whole brain radiotherapy in patients with brain metastases

Bolanle Adegboyega^1*^, Adedayo Joseph^1^, Adewumi Alabi^1^, John Omomila^1^, Lindokuhle M. Ngema^2*^, Victoria Ainsworth^2,3^, Jennifer Chin^2^, Moses Evbuomwam^4^, Wilfred Ngwa^2^

*^1^NSIA-LUTH Cancer Centre, Lagos University Teaching Hospital, Lagos, Nigeria*

*^2^Johns Hopkins Medicine, Sydney Kimmel Comprehensive Cancer Centre, Baltimore, MD 21218, USA*

*^3^Department of Physics, University of Massachusetts Lowell, Lowell, MA 01854, USA*

*^4^Department of Radiation Oncology, University of Iowa Hospitals and Clinics, Iowa City, IA 52242, USA*

***Corresponding Author(s):**

Dr. Bolanle Adegboyega; **E**: [abecee2001@yahoo.com](mailto:abecee2001@yahoo.com)

Lindokuhle M. Ngema; **E**: [845407@students.wits.ac.za](mailto:845407@students.wits.ac.za)

**Table S1:** Brain metastasis variable key

|  | | | |
| --- | --- | --- | --- |
| **Column** | **Variable** | **Description** | **Codes\Values** |
|  |  | **DEMOGRAPHICS** |  |
| A | PID | Patients ID |  |
| B | AGE | Age | Yrs |
| C | SEX | Sex | Male = 0; Female = 1 |
| D | POR | Place of Residence | Urban = 0; Rural = 1 |
| E | REL | Religion | Christian =0; Islam =1; Others = 2 |
| F | EDU | Level of Education | Secondary =0; Postsecondary =1 |
| G | MS | Marital Status | Single = 0; Married = 1; Divorce = 2; Widow = 3 |
| H | ES | Employment Status | Employed = 0; Unemployed =1; Self-employed =2; Retired = 3 |
| I | OCC | Occupation | Business (Business / Trader / Printer / Insurance marketer / Beautician / Sale Rep) = 0;  Government Worker (Civil servant / Community Relation Officer) = 1;  Non-academia (Clergy / Actor / Actress / Contractor) = 2;  Other jobs (Retired / Unemployed / Nil / None / Housewife / Pensioner) = 3;  Academia (Teacher, Lecturers, Students, Engineer) = 4 |
| J | PT | Primary Tumor | Anaplastic Thyroid = 0; Breast = 1  Carcinoid Tumor = 2; Cup = 3  GI = 4; Head and Neck = 5  LT Thigh = 6; Lungs = 7  Parotid = 8; Prostate = 9  Renal = 10 |
| K | SIRR | Site Irradiation | Brain = 0 |
| L | ECPS | ECOG Performance Status | KPS 90-100 = 0; KPS 70-80 =1; KPS 50-60 =2; KPS 30-40 =3; KPS 10-20 = 4; KPS 0 =5 |
| M | PRIOR | Prior Radiotherapy | No = 0; Yes =1 |
| N | RDG | Radiation Dose in GY | 20 = 0; 30 = 1 |
| O | RDF | Radiation Dose in Fraction | 5 = 0; 10 = 1 |
| P | TSD | Time since diagnosis of metastatic brain disease (days) | 1 week = 7 days  1 month = 30 days |
| Q | COM | Co-morbidities | Absent = 0; Present = 1 |
| R | MEDS | Medication Used | Steroids = 0; Analgesic =1; Analgesic/Steroids = 2 |
|  |  |  |  |
|  |  |  |  |
|  |  | **EORTC QLQ-C15-PAL** |  |
| S | PAL1 | Do you have any trouble taking a short walk outside of the house? | Not at all = 1; A little = 2; Quite a Bit =3; Very much = 4 |
| T | PAL2 | Do you need to stay in bed or a chair during the day? | Not at all = 1; A little = 2; Quite a Bit =3; Very much = 4 |
| U | PAL3 | Do you need help with eating, dressing, washing yourself or using the toilet? | Not at all = 1; A little = 2; Quite a Bit =3; Very much = 4 |
| V | PAL4 | Were you short of breath? | Not at all = 1; A little = 2; Quite a Bit =3; Very much = 4 |
| W | PAL5 | Have you had pain? | Not at all = 1; A little = 2; Quite a Bit =3; Very much = 4 |
| X | PAL6 | Have you had trouble sleeping? | Not at all = 1; A little = 2; Quite a Bit =3; Very much = 4 |
| Y | PAL7 | Have you felt weak? | Not at all = 1; A little = 2; Quite a Bit =3; Very much = 4 |
| Z | PAL8 | Have you lacked appetite? | Not at all = 1; A little = 2; Quite a Bit =3; Very much = 4 |
| AA | PAL9 | Have you felt nauseated? | Not at all = 1; A little = 2; Quite a Bit =3; Very much = 4 |
| AB | PAL10 | Have you been constipated? | Not at all = 1; A little = 2; Quite a Bit =3; Very much = 4 |
| AC | PAL11 | Were you tired? | Not at all = 1; A little = 2; Quite a Bit =3; Very much = 4 |
| AD | PAL12 | Did pain interfere with your daily activities? | Not at all = 1; A little = 2; Quite a Bit =3; Very much = 4 |
| AE | PAL13 | Did you feel tense? | Not at all = 1; A little = 2; Quite a Bit =3; Very much = 4 |
| AF | PAL14 | Did you feel Depressed? | Not at all = 1; A little = 2; Quite a Bit =3; Very much = 4 |
| AG | PAL15 | How would you rate your overall quality of life during the past week? | Very poor =1; Excellent=7 |
|  |  | **EORTC QLQ-BN20** |  |
| AH | BN31 | Did you feel uncertain about the future? | Not at all = 1; A little = 2; Quite a Bit =3; Very much = 4 |
| AI | BN32 | Did you feel you had setbacks in your condition? | Not at all = 1; A little = 2; Quite a Bit =3; Very much = 4 |
| AJ | BN33 | Were you concerned about disruption of family life? | Not at all = 1; A little = 2; Quite a Bit =3; Very much = 4 |
| AK | BN34 | Did you have headaches? | Not at all = 1; A little = 2; Quite a Bit =3; Very much = 4 |
| AL | BN35 | Did you outlook on the future worsen? | Not at all = 1; A little = 2; Quite a Bit =3; Very much = 4 |
| AM | BN36 | Did you have double vision? | Not at all = 1; A little = 2; Quite a Bit =3; Very much = 4 |
| AN | BN37 | Was your vision blurred? | Not at all = 1; A little = 2; Quite a Bit =3; Very much = 4 |
| AO | BN38 | Did you have difficulty reading because of your vision? | Not at all = 1; A little = 2; Quite a Bit =3; Very much = 4 |
| AP | BN39 | Did you have seizures? | Not at all = 1; A little = 2; Quite a Bit =3; Very much = 4 |
| AQ | BN40 | Did you have weakness on one side of your body? | Not at all = 1; A little = 2; Quite a Bit =3; Very much = 4 |
| AR | BN41 | Did you have trouble finding the right words to express yourself? | Not at all = 1; A little = 2; Quite a Bit =3; Very much = 4 |
| AS | BN42 | Did you have difficulty speaking? | Not at all = 1; A little = 2; Quite a Bit =3; Very much = 4 |
| AT | BN43 | Did you have trouble communicating your thoughts? | Not at all = 1; A little = 2; Quite a Bit =3; Very much = 4 |
| AU | BN44 | Did you feel drowsy during the daytime? | Not at all = 1; A little = 2; Quite a Bit =3; Very much = 4 |
| AV | BN45 | Did you have trouble with your coordination? | Not at all = 1; A little = 2; Quite a Bit =3; Very much = 4 |
| AW | BN46 | Did hair loss bother you? | Not at all = 1; A little = 2; Quite a Bit =3; Very much = 4 |
| AX | BN47 | Did itching of your skin bother you? | Not at all = 1; A little = 2; Quite a Bit =3; Very much = 4 |
| AY | BN48 | Did you have weakness of both legs? | Not at all = 1; A little = 2; Quite a Bit =3; Very much = 4 |
| AZ | BN49 | Did you feel unsteady on your feet? | Not at all = 1; A little = 2; Quite a Bit =3; Very much = 4 |
| BA | BN50 | Did you have trouble controlling your bladder? | Not at all = 1; A little = 2; Quite a Bit =3; Very much = 4 |
|  |  |  |  |

**SHEET CODES**

R0 = BASELINE

R1 = 7 DAYS AFTER TREATMENT

R2 = 30 DAYS AFTER TREATMENT

R3 = 90 DAYS AFTER TREATMENT

R4 = 180 DAYS AFTER TREATMENT
