# Supplementary material for: Patient reported outcomes following whole brain radiotherapy in patients with brain metastases in NSIA-LUTH Cancer Center
Source: BMC Cancer. 2023 Dec 14;23:1233. doi: 10.1186/s12885-023-11675-8 (PMC10722749; doi:10.1186/s12885-023-11675-8)
Supplement: Supplementary file 2 — Additional file 2. Score transformation procedure. [file 12885_2023_11675_MOESM2_ESM.docx]

Patient reported outcomes following whole brain radiotherapy in patients with brain metastases

Bolanle Adegboyega^1*^, Adedayo Joseph^1^, Adewumi Alabi^1^, John Omomila^1^, Lindokuhle M. Ngema^2*^, Victoria Ainsworth^2,3^, Jennifer Chin^2^, Moses Evbuomwam^4^, Wilfred Ngwa^2^

*^1^NSIA-LUTH Cancer Centre, Lagos University Teaching Hospital, Lagos, Nigeria*

*^2^Johns Hopkins Medicine, Sydney Kimmel Comprehensive Cancer Centre, Baltimore, MD 21218, USA*

*^3^Department of Physics, University of Massachusetts Lowell, Lowell, MA 01854, USA*

*^4^Department of Radiation Oncology, University of Iowa Hospitals and Clinics, Iowa City, IA 52242, USA*

***Corresponding Author(s):**

Dr. Bolanle Adegboyega; **E**: [abecee2001@yahoo.com](mailto:abecee2001@yahoo.com)

Lindokuhle M. Ngema; **E**: [845407@students.wits.ac.za](mailto:845407@students.wits.ac.za)

**SCORE TRANSFORMATION PROCEDURE**

**(A) EORTC QLQ-C15-PAL**

- 2-multi-item scales (physical and emotional functioning)
- 2-multi-item scales (fatigue and pain)
- 5-single-item scales (dyspnea, insomnia, appetite loss, nausea/vomiting, and constipation)
- 1-overall QOL (global health status)

1. Function Scales
   1. Physical functioning (PF) = (Q 1 + Q2 + Q3)
   2. Emotional functioning (EF) = (Q13 + Q14)
2. Symptom Scales
   1. Fatigue (FA) = (Q7 + Q11)
   2. Pain (PA) = (Q 5 + Q12)
   3. Dyspnea (DY) = (Q4)
   4. Insomnia (SL) = (Q6)
   5. Appetite loss (AP) = (Q 8)
   6. Nausea/vomiting (NV)= (Q9)
   7. Constipation (CO) = (Q10)
3. Global health status (quality of life, QoL) = (Q 15)


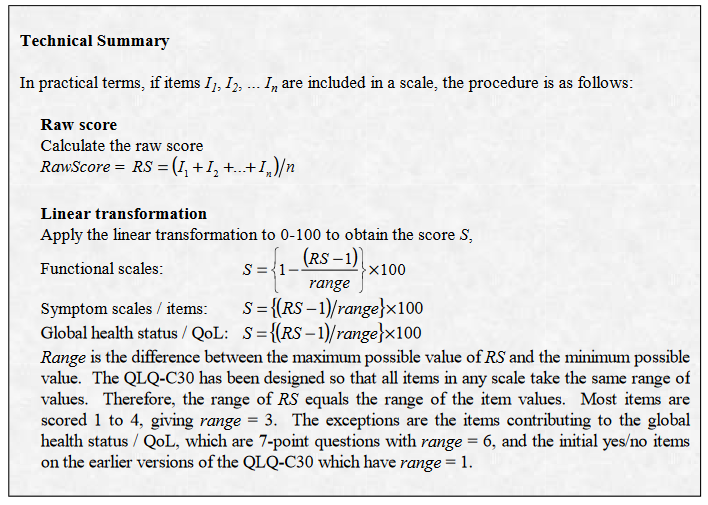


All QLQ-C15-PAL scale scores were linearly transformed, and the resultant scores ranged from 0 to 100

**(B) EORTC QLQ-BN20**

- 4-multi-item scales (future uncertainty)
- 3-multi-item scales (visual disorder, motor dysfunction, and communication deficit)
- 7-single-item scales (headache, seizure, drowsiness, hair loss, itchy skin, weakness of leg, and bladder control)

1. Symptom Scales

**Multi-item scales**

- 1. Future uncertainty = (Q31 + Q32 + Q33 + Q35)
  2. Visual disorders = (Q36 + Q37 + Q38)
  3. Motor dysfunction = (Q40 + Q45 + Q49)
  4. Communication deficit = (Q41 + Q42 + Q43)

**Single-item scales**

- 1. Headache = (Q34)
  2. Seizures = (Q39)
  3. Drowsiness = (Q44)
  4. Hair loss = (Q46)
  5. Pruritus or Itchy skin = (Q47)
  6. Weakness of both leg = (Q48)
  7. Difficulties with bladder control = (Q50)

The self-assessed scale scores ranging from 1–4 is then linearly converted to a 0–100 scale, with higher scores indicating more severe symptoms. In the case of functions, higher scores indicate better function.
